# Printability, Mechanical and Thermal Properties of Poly(3-Hydroxybutyrate)-Poly(Lactic Acid)-Plasticizer Blends for Three-Dimensional (3D) Printing

**DOI:** 10.3390/ma13214736

**Published:** 2020-10-23

**Authors:** Soňa Kontárová, Radek Přikryl, Veronika Melčová, Přemysl Menčík, Matyáš Horálek, Silvestr Figalla, Roderik Plavec, Jozef Feranc, Jiří Sadílek, Aneta Pospíšilová

**Affiliations:** 1 Institute of Materials Chemistry, Faculty of Chemistry, Brno University of Technology, Purkyňova 464/118, 612 00 Brno, Czech Republic; prikryl@fch.vut.cz (R.P.); xcmelcova@fch.vut.cz (V.M.); mencik@fch.vut.cz (P.M.); xchoralek@fch.vut.cz (M.H.); xcfigallas@fch.vut.cz (S.F.); xcpospisilovaan@fch.vut.cz (A.P.); 2 Institute of Natural and Synthetic Polymers, Faculty of Chemical and Food Technology, Slovak University of Technology in Bratislava, Radlinského 9, 812 37 Bratislava, Slovakia; roderik.plavec@stuba.sk (R.P.); jozef.feranc@stuba.sk (J.F.); 3 Polymer Institute Brno, Tkalcovská 36/2, 602 00 Brno, Czech Republic; jiri.sadilek@polymer.cz

**Keywords:** plasticizers, poly(hydroxybutyrate)-poly(lactic), 3D printing, printability, citrates, poly(ethylene glycol) PEG, biodegradable polymeric blends

## Abstract

This paper investigates the effect of plasticizer structure on especially the printability and mechanical and thermal properties of poly(3-hydroxybutyrate)-poly(lactic acid)-plasticizer biodegradable blends. Three plasticizers, acetyl tris(2-ethylhexyl) citrate, tris(2-ethylhexyl) citrate, and poly(ethylene glycol)bis(2-ethylhexanoate), were first checked whether they were miscible with poly(3-hydroxybutyrate)-poly(lactic acid) (PHB-PLA) blends using a kneading machine. PHB-PLA-plasticizer blends of 60-25-15 (wt.%) were then prepared using a corotating meshing twin-screw extruder, and a single screw extruder was used for filament preparation for further three-dimensional (3D) fused deposition modeling (FDM) printing. These innovative eco-friendly PHB-PLA-plasticizer blends were created with a majority of PHB, and therefore, poor mechanical properties and thermal properties of neat PHB-PLA blends were improved by adding appropriate plasticizer. The plasticizer also influences the printability of blends, which was investigated, based on our new specific printability tests developed for the optimization of printing conditions (especially printing temperature). Three-dimensional printed test samples were used for heat deflection temperature measurements and Charpy and tensile-impact tests. Plasticizer migration was also investigated. The macrostructure of 3D printed samples was observed using an optical microscope to check the printing quality and printing conditions. Tensile tests of 3D printed samples (dogbones), as well as extruded filaments, showed that measured elongation at break raised, from 21% for non-plasticized PHB-PLA reference blends to 84% for some plasticized blends in the form of filaments and from 10% (reference) to 32% for plasticized blends in the form of printed dogbones. Measurements of thermal properties (using modulated differential scanning calorimetry and oscillation rheometry) also confirmed the plasticizing effect on blends. The thermal and mechanical properties of PHB-PLA blends were improved by the addition of appropriate plasticizer. In contrast, the printability of the PHB-PLA reference seems to be slightly better than the printability of the plasticized blends.

## 1. Introduction

Environmental issues caused by plastics have induced rising concern towards “biopolymers”, polymers that are biodegradable or bio-based or can be both. Bioplastics made from biopolymers already serve as alternatives for many petroleum-based commodities and have a predicted global production capacity of around 2.62 million tons for 2023. Researchers are trying to seek bioplastics which have comparable or even better properties to those of petroleum-based plastics. Bioplastics are already used in an increasing number of branches, e.g., packaging, catering products, agriculture, coatings and adhesives, toys, textiles, electronics, automotive, building and construction, medical applications, and many other sectors. Biopolymers PLA (poly(lactic acid)) and PHAs (polyhydroxyalkanoates) are generally well-researched and are the prime leaders of biopolymers production capacity growth [1,2,3,4,5,6,7].

PHAs are bio-based and biodegradable linear polyesters and copolymers with a broad range of mechanical and physical properties, which depend on PHA chemical composition. The production capacity of PHAs is expected to triple from 2018 to 2023 [7]. PHAs are embedded as intracellular carbon and energy reserve granules in different Gram-positive and Gram-negative bacteria from a number of substrates, including sugars and fatty acids [8]. The most common presence of short-chain-length PHAs (scl-PHA) are those with 3–5 carbon atoms per unit [8,9]. The subject of our study is poly(3-hydroxybutyrate) (PHB), the most researched and widely used representative of the PHAs group. The advantage of PHB is the possibility of its preparation also from waste cooking oil so it does not require agronomical feedstock [10,11].

Poly(lactic acid) (PLA), ‒[CH(CH_3_)COO]_n_‒, is a highly transparent, glossy, colorless, and rigid thermoplastic material with great barrier properties. This aliphatic polyester is synthesized from lactic acid, which is produced by the fermentation of renewable resources and is available in the form of high-performance PLA grades that are a good replacement for PP (polypropylene), PS (polystyrene), and ABS (acrylonitrile butadiene styrene) in many demanding applications [7,12,13,14,15,16]. The ratio between d and l enantiomers influences the properties of PLA. Among the disadvantages of PLA are their low heat distortion temperatures (HDTs): softening above 60 °C. A disadvantage is also its incapability of fast biodegrading at ambient temperature [13]. The tensile properties of PLA can be various and are influenced by its degree of crystallinity and whether it is annealed or oriented [14,17].

Due to nontoxicity, biodegradability, and biocompatibility, these PHB (poly(3-hydroxybutyrate)) and PLA blends can be useful together with three-dimensional (3D) printing technologies, especially for biomedicine (implants, scaffolds), art, archeology (replicas), and for prototyping and manufacture of reserve parts for the automotive industry, airplanes, etc. [12,18,19,20]. While PLA has great printability and is commonly used in 3D printing, printability of pure PHB (synthesized by bacterial fermentation) is more complicated due to its high crystallinity. It is related mostly to warping (overall shrinkage is caused not only by thermal shrinkage but in addition also crystallization shrinkage occurring during melt cooling). The principal disadvantage for the printing of PHB is that its melting temperature (around 170–180 °C) is too close to the degradation temperature. Thus, its printability is limited by this relatively small processing window. Filaments of PLA-PHA polymeric blends are already used for 3D printing also in industry, and its printability is good due to the majority of PLA [21,22].

In this study, fused deposition modeling (FDM) technology is used. This technology is very productive also while using low-cost 3D printers. A plastic filament (in our study PHB-PLA-plasticizer filament) used in the FDM method is pushed through an extrusion nozzle heated to a particular temperature and is melted and deposited on the printing bed in the desired conformation. A part of the FDM method is the software that compiles an STL (stereolithography) or CAD (computer-aided design) file, mathematically slicing and orienting the model for the building process [23]. This method is used in our work for the preparation of 3D printed dogbones (double-paddle shaped testing samples) from PHB-PLA-plasticizer material, which are further used for tensile tests, HDT measurements, Charpy, and tensile-impact tests. Before the printing of a test specimen, the printability had to be tested to setup the best printing conditions (especially printing temperature). The warping problem can occur especially during the printing of large parts because the internal temperature of a printed object during printing tends to vary. PHB-PLA-plasticizer materials are printed at high extrusion nozzle temperatures (180‒195 °C) onto the printing bed, which is at room temperature. An integrated fan is used for cooling and for quick solidification of the material. The shrinkage which is usually caused by this situation can lead to deformation through warping. As a result, detachment from the printing bed and the destruction of products usually occur [24]. In general, FDM “printability” is not well defined and there are not standard specifications describing printability, probably due to the many types of printers, methods, and printing parameters that can influence the printability of the final product. The printability of our PHB-PLA-plasticizer blends and warping were tested according to practical tests designed by our research group. These tests are focused on determining the appropriate temperature of the extrusion nozzle, especially the temperature at which geometrical shapes are printed with the best quality (designed temperature tower test, see Materials and Methods) and with minimal warping (designed warping test specimen). These newly designed tests are applicable for all materials and FDM printers and can be useful tools to set up the best temperature of the extrusion nozzle for the printing of the desired products.

To take advantage of PLA and PHB as biocompatible and biodegradable alternatives to customary polymers in industry, some disadvantages must be overcome. Toughness, fragility, brittleness, and poor processing properties of neat PHB can be exceeded by blending with poly(d,l-lactic acid). This amorphous polymer can reduce the crystallinity of PHB and so expand the range of applications of PHB [3,25]. Still, neat PHB-PLA blends are brittle and stiff materials, have poor mechanical properties, and due to thermal degradation near to its melting point also have limited processability [26]. The addition of plasticizer can improve poor mechanical properties, especially poor ductility. These non-volatile low molecular weight compounds, used as additives in the polymer industry, are able to lower the melting temperature (*T*_m_), the glass transition temperature (*T*_g_), and improve the flexibility and the processability of biopolymers [27,28]. The addition of plasticizers can influence a lot of properties: they can decrease the tension of deformation, density, hardness, viscosity, they can increase resistance to fracture, dielectric constant, the polymer chain flexibility, and they can influence optical clarity, the degree of crystallinity, electric conductivity, the resistance to biological degradation, and fire conductivity [27,29]. A lot of plasticizers have already been investigated, such as various monomeric esters of adipic, phosphoric, phthalic, citric, ricinoleic and sebacic acids, chlorinated paraffin, and low-molecular-weight polyesters [27,28].

Two commercial and synthesized eco-friendly primary plasticizers that are based on esters of citric acid (acetyl tris(2-ethylhexyl) citrate, tris(2-ethylhexyl) citrate), and poly(ethylene glycol) bis(2-ethylhexanoate) were used in this current study. The goal was to improve the poor mechanical properties of neat PHB-PLA blends (with the majority of PHB) by adding an appropriate plasticizer and to make new types of eco-friendly PHB-PLA-plasticizer blends which are suitable for later application as scaffolds in biomedicine. Our previous research [30] was concerned with PHB-PLA-plasticizer, where different plasticizers, including tributyl citrate and acetyl tributyl citrate, were used. In this current study, also printability, which is necessary to be well explored for the printing of scaffold and other products, is investigated. At the beginning, the miscibility of new plasticizers with PHB-PLA blends in the weight ratio (wt.%) of 85polymer-15plasticizer was checked using a kneading machine. The 60PHB-25PLA-15 plasticizer blends (in wt.%) were then prepared by corotating meshing twin-screw extruder, and next, a single screw extruder was used for filaments preparation (for further 3D printing). The printability of 3D samples printed from these blends was judged according to our newly designed tests. The printability test “Temperature tower” could reveal the best nozzle printing temperature for each PHB-PLA-plasticizer blend and also compared the influence of particular plasticizers on the printability of geometrical shapes. The printability test “Warping specimen” revealed the influence of particular plasticizers on warping. In addition, the best nozzle printing temperatures, at which the warping during printing was minimal, were found. Thus, the optimization of printing conditions via printing tests allowed us to print PHB-PLA-plasticizer test specimens (for further mechanical, thermal, and optical tests) with the best obtainable printing quality. The printing quality was finally checked using the 3DBenchy test. The mechanical properties of these plasticized blends were further estimated using tensile tests of filaments as well as 3D printed dogbones, which were printed at optimized conditions after printability tests. 3D printed dogbones were used also for Charpy and tensile-impact tests. The migration of plasticizers from blends, which is the principal disadvantage of external plasticizing (especially in the case of linear plasticizer’s structure compared to branched [31,32,33,34,35,36,37]) was also investigated by exposing blends to 110 °C in the drying oven. Modulated differential scanning calorimetry (MDSC) was used to investigate the thermal properties of our blends, as well as the HDT (with 3D printed test specimens), and oscillation rheometry (RPA 2000) measurements. The macrostructure of 3D printed samples, printed at the conditions adjusted according to printability tests, and their printing quality was checked using an optical microscope.

Papers engaged in the improvement of the thermal and mechanical properties of PLA-PHB blends using plasticizers have already been published but always with a PLA majority. Among the investigated plasticizers are, e.g., oligomeric lactic acid [38], acetyl tributyl citrate and poly(ethylene glycol) [26,39], polyester plasticizer (Lapol 108) [3]. Papers dealing with the printability of PLA or PHB or different biobased polymer are even rarer. Diederichs et al. [40] investigated how to improve the printability of biobased poly(trimethylene terephthalate) (PTT) with additives such as chain extenders (CEs) and impact modifiers. They were able to prepare 3D printed complete and warpage-free samples because of the modifications made in processing and printing parameters, and with the assistance of additives. The 3D printing quality of seven PLA grades analyzed based on MFI (melt flow index), density, SEM (scanning electron microscopy), and DSC (differential scanning calorimetry) was investigated by Wang et al. [41]. They also found that commercial PLA3D850 is a promising material for 3D printing in the case that PHB is used as a blending partner (80-20 PLA-PHB by weight) and that this blending leads to a fully biodegradable material with good processability and no need for annealing.

Our research describes the influence of three plasticizers on printability and the mechanical and thermal properties of plasticized PHB-PLA blends, with a majority of PHB. Research of our blends with acetyl tris(2-ethylhexyl) citrate, tris(2-ethylhexyl) citrate, and poly(ethylene glycol) plasticizers and the section describing the “printability” of PHB-PLA-plasticizer blends has not been published yet, and thus, it is unique in this context. Our printability tests developed for the optimization of printing conditions could be useful also for the case of other materials and types of printers. Our PHB-PLA-plasticizer blends are suitable for 3D printing, biomedical applications and prototyping. The advantage of blends with a majority of PHB is their ability to be well compostable and biodegradable.

## 2. Materials and Methods

### 2.1. Materials

Poly-3-hydroxybutyrate (PHB Biomer^®^, *ρ* = 1.23 g∙cm^−3^, *M*_w_ = 410,000 g·mol^−1^) was provided by Biomer Company (Krailling, Germany) in the form of white powder [42]. In our study amorphous poly(d,l-lactic acid) (PLA) with *T_g_* around 60 °C is used. Amorphous PLA was chosen for its ability to reduce the overall crystallinity content in our PHB-PLA-plasticizers blends, which are further used for 3D printing. Amorphous PLA granules (Ingeo™ 4060D, *M*_w_ = 180 000 g·mol^−1^, *T*_g_ = 55–60 °C, *ρ* = 1.24 g∙cm^−3^) were supplied by NatureWorks LLC (Minnetonka, MN, USA) Company [43].

**Figure 1:**
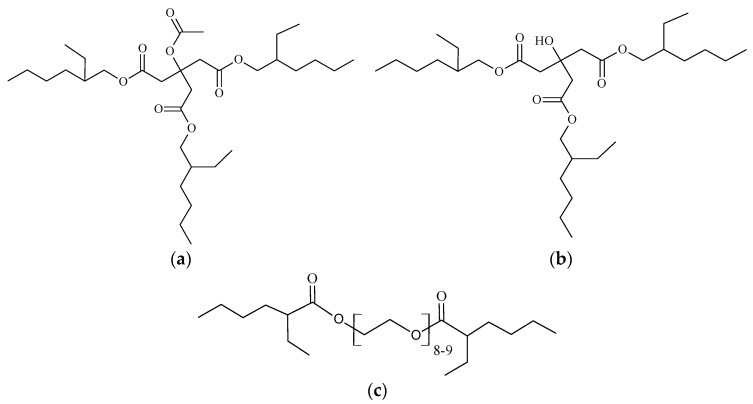
(**a**) Plasticizer A2-EH structure; (**b**) plasticizer C2-EH expected structure; and (**c**) plasticizer PEG-2-EH structure.

Monomeric commercial plasticizer used in this research is based on the ester of citric acid (acetyl tris(2-ethylhexyl) citrate) and is provided by Jungbunzlauer Suisse AG (Basel, Switzerland), with the Citrofol^®^ trademark [44]. An additional monomeric plasticizer based on the ester of citric acid (tris(2-ethylhexyl) citrate) was synthesized for this study. Another commercial plasticizer, poly(ethylene glycol) bis(2-ethylhexanoate), was provided by Merck KGaA (Darmstadt, Germany) [45]. The plasticizers described by commercial and chemical names, molecular weights, and labels can be seen in Table 1. Their structure can be seen in Figure 1.

### 2.2. Plasticizers Miscibility with PLA-PHB Polymers

60PHB-25PLA-15 plasticizer samples (in wt.%) and a non-plasticized 70PHB-30 PLA reference sample were kneaded using a kneading machine and according to conditions described in our previous work [30]. There were only small changes in the process: the weights of samples were 50 g (before the kneading). Sample dosing (PHB-PLA mixture) into the Brabender machine (Brabender GmbH & Co. KG, Duisburg, Germany) took 1 min, and then, the mixture was kneaded for another 2 min before plasticizer was added. The kneading process continued for the following 6 min. The total kneading time of PHB-PLA-plasticizer samples as well as PHB-PLA reference was 9 min.

### 2.3. Preparation of Plasticized PHB-PLA Blends

The 70PHB-30PLA blends (in wt.%) (reference), as well as the 60PHB-25PLA-15 plasticizer blends (in wt.%), were extruded using two extruders according to conditions described in our previous work [30]. Citrofol^®^ (A2-EH), C2-EH, and PEG-2-EH were used as plasticizers stepwise. Conditions and process details were the same as in our previous work, except the total weights of each sample for extrusion, which were 1000 g for plasticized samples and 850 g for reference. Filaments with well-defined thicknesses and diameters were prepared for further 3D printing.

### 2.4. 3D Printing

The dogbone samples prepared for further tensile tests and sample bars (80 × 10 × 4) mm prepared for further HDT measurements, Charpy and tensile-impact strength tests and for the observations using optical microscopy were first designed using AutoCAD (ver. 2018, Autodesk Inc., San Rafael, CA, USA) and Slic3r (software version 1.3.0, free software, developed by Alessandro Ranellucci). These programs were used for 3D virtual modeling and mathematical slicing. The 3D model for the printing of dogbone specimens was designed by our group and is described in detail in our previous work [30]. Sample bars were also designed using Slic3r and uploaded to the printer. Their 3D model was generated simply by only perimeters (100%) using no infill.

All test specimens were printed on a PRUSA i3 MK3 3D printer (Prusa Research s.r.o., Praha, Czech Republic) using FDM technology from PHB-PLA-plasticizer filaments with a defined diameter, as mentioned above. The dogbones and sample bars were printed at 180–195 °C (195 °C during the printing of first layers for better adhesion to the printing bed), without additional air cooling and bed heating. Specific printing temperatures for each PHB-PLA-plasticizer sample were selected according to our printability test results (described below), where the temperature tower, the warping, and 3DBenchy tests were examined. Other basic printing parameters for testing samples can be seen in Table 2. Three-dimensional printing was executed approximately three months after preparation of the filaments.

### 2.5. Printability Tests

The printability tests were designed for the optimization of printing conditions, in order to improve the structural homogeneity and thus mechanical properties of our samples. The printing temperature was optimized via temperature tower tests and warping tests designed by our team. Final verification of adjusted printing conditions proceeded via the Benchy test.

#### 2.5.1. Temperature Tower

**Figure 2:**
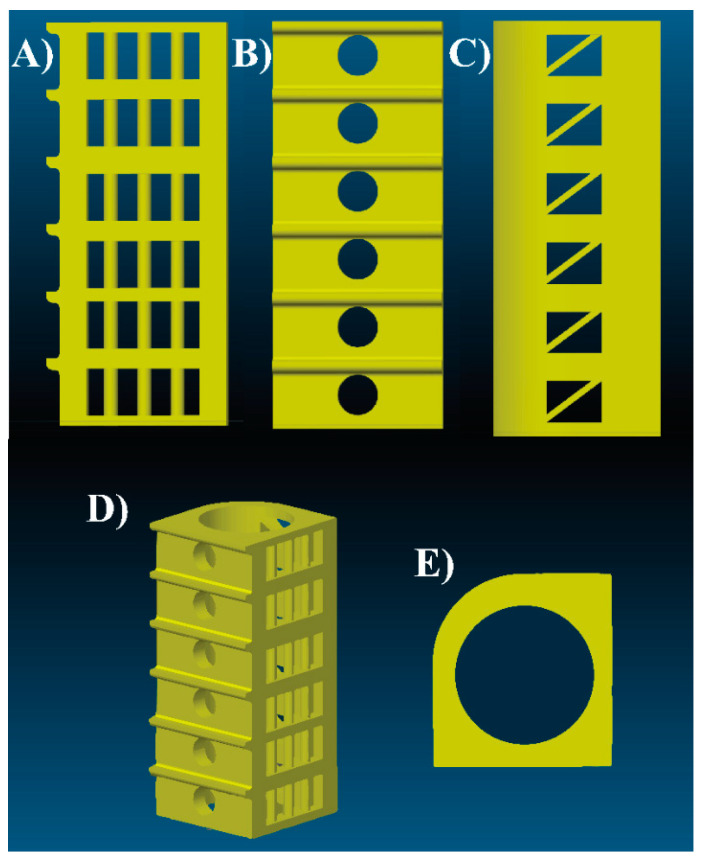
Temperature tower model: (**A**–**C**) lateral views, (**D**) axonometric view, (**E**) top view [46].

One of the most important 3D printing parameters is the temperature of the printing nozzle. The printability temperature interval for each PHB-PLA-plasticizer blend was examined via a test specimen temperature tower, designed by our team [46], with the help of Autodesk Fusion 360 software (ver. 2018, Autodesk Inc., San Rafael, CA, USA). The temperature tower (see Figure 2) was designed regarding the printability speed, amount of used material, and number of various geometric elements. The temperature tower consists of six identical floors and each of them contains eight different geometric elements. The dimensions of the temperature tower are approximately (60 × 25 × 25) mm. It is possible to set up individual printing temperatures for each floor. Three temperature towers were 3D printed in the case of each PHB-PLA-plasticizer blend, first in the temperature interval 220–195 °C (220 °C the first floor, each following floor 5 °C less than the previous floor, 195 °C the last floor), second in the temperature interval 195–170 °C, and third in the temperature interval 180–155 °C. Ideal temperatures for each PHB-PLA-plasticizer blend were then visually evaluated by the comparison of floors one to another and by the measurement of dimensional variations of printed geometric elements compared to the software model. Among the observed geometric elements, there were colonnade and bridges between floors (Figure 2A), horizontal circle holes and overhangs between floors (Figure 2B), diagonal crossbar and rectangle holes (Figure 2C), roundness of the wall (Figure 2D,E), and vertical circle hole and eventual stringing in it (Figure 2E). In addition, the color of each floor was checked and compared. The darker coloration of some floors, especially during the printing with higher nozzle temperatures, suggested material degradation and inapplicable nozzle temperature. The temperature tower provides information about the range of applicable printability temperatures and ideal temperature for a particular material, together with warping test results.

#### 2.5.2. Warping

**Figure 3:**
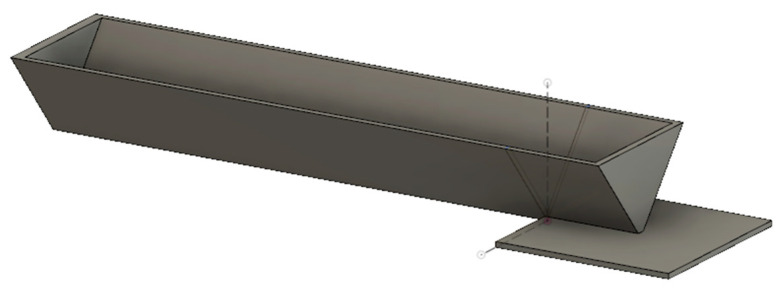
Three-dimensional model of designed warping test specimen [46].

A standardized method, enabling easy, quick, and repetitive measurements of warping of 3D printed materials is not currently available. Hence, our team designed the test specimen, which fulfills the demands for repeatable warping measurements. The warping specimen consists of a square platform (20 × 20) mm and a stuck beam (60 × 10 × 10) mm, which adhere to the bed only via one peripheral wall [46] (Figure 3).

The warping during the printing of the test specimen causes the lifting of a stuck beam at the opposite end from the square platform. The first detachment of a stuck beam from the bed occurred during the printing of the warping specimen, and the height of the layer (value of *z*-axis during printing subtracted from the printer) was observed and recorded. The designed total height of the warping test specimen (10 mm) was then divided by the recorded value, and in this way, the warping coefficient was calculated (Equation (1)). The later the negative effect of warping starts to manifest, the lower the warping coefficient and therefore the material is more printable.

(1) $$\mathrm{warping}\;\mathrm{coefficient}\;=\;\frac{\mathrm{designed}\;\mathrm{height}\;\mathrm{of}\;\mathrm{warping}\;\mathrm{test}\;\mathrm{specimen}}{\mathrm{height}\;\mathrm{of}\;\mathrm{warping}\;\mathrm{test}\;\mathrm{specimen}\;\mathrm{achieved}\;\mathrm{during}\;\mathrm{printing}}$$

Several applicable temperatures, based on the temperature tower results, were selected for 3D printing of warping specimens printed from each PHB-PLA-plasticizer filament. The temperatures, for which the warping coefficient was the lowest, were then applied for 3D printing of test specimens from each PHB-PLA-plasticizer filament. These test specimens were subjected to tensile tests, HDT, Charpy and tensile-impact strength tests, and observations using optical microscopy. The first layer temperature during the 3D printing was always set to 195 °C for better adhesion to the printing bed. This value was experimentally established as the best for PHB-PLA-plasticizer filaments [46].

#### 2.5.3. 3D Benchy

The 3D Benchies were printed for each PHB-PLA-plasticizer filament at printing temperatures optimized via the temperature tower and warping tests. Resultant printing temperatures varied in the range of 180–195 °C (the first layers were printed at 195 °C). Printed 3D Benchies were then visually evaluated [47], and thus, the adjusted printing conditions were revised.

### 2.6. Modulated Differential Scanning Calorimetry—Thermal Characterization

The combination of modulated and conventional differential scanning calorimetry (MDSC and DSC, respectively) was used for the thermal properties measurements of prepared PHB-PLA-plasticizer and PHB-PLA reference samples. The measurements were performed according to ASTM D 3417-99 standard specification [48]. Process details and measurements conditions were the same as in our previous work; for further details please see [30].

### 2.7. Oscillation Rheometry—Thermal Characterization

The oscillation rheometer RPA 2000 (Alpha Technologies GmbH, Germany, Schwabach) with parallel biconical chambers geometry was used for the rheology measurements according to the methodology tailored for bioplastics [49]. All PHB-PLA-plasticizer samples and PHB-PLA reference were subjected to a 10 min timed test at 180 °C. The used frequency was 0.833 Hz (50 oscillations per minute). The amplitude of deformation was chosen to be 30% in order to simulate the conditions during processing. The samples for rheology measurements were in the form of granules prepared from PHB-PLA-plasticizer filaments. The tests were carried out for 7 and 40 days after the preparation of the pellets.

For the evaluation of the degradation rate under thermal and shear loading, the relative complex viscosity was calculated for each sample according to the formula:

(2) $$\eta_{rel}^{*}\left(t\right)\;=\;\frac{\eta^{*}\left(t\right)}{\eta^{*}\left(0\right)}$$

where $\eta_{rel}^{*}\left(t\right)$ is relative complex viscosity in time *t*, $\eta^{*}\left(t\right)$ is complex viscosity in time *t*, and $\eta^{*}\left(0\right)$ is complex viscosity at the start of the timed test.

### 2.8. GPC–Gel Permeation Chromatography

MW (Molecular Weights) averages and polydispersity indexes for both neat and modified polymers and blends were measured using gel permeation chromatography (GPC). The measurements were performed using an Agilent Technologies 1100 Series instrument equipped with an isocratic pump, fraction collector, and autosampler. PLgel 10 μm MIXED-B column was thermostated to 30 °C with chloroform as the eluent at the flow rate of 0.1 mL/min. Linear polystyrene standards with narrow distribution were used to gain the calibration curve (10 points in calibration). The instrument is equipped with a refractive index detector. For the measurement, 5 mg of sample was used.

### 2.9. HDT—Heat Deflection Temperature

A custom-made apparatus for the HDT measurements of plastics under flexural load in flatwise position was used for the testing of 3D printed PHB-PLA-plasticizer samples, according to CSN EN ISO 75 standard specifications [50]. Three-dimensional printed sample bars, (their 3D model was formed from perimeters only) with (80 × 10 × 4) mm dimensions, were immersed under load in a heat-transfer medium (silicon oil) provided with the means of raising the temperature by (2 ± 0.2) °C/min. The load applied to the specimen center to give the maximum fiber stresses was 1.82 MPa and 0.455 MPa (Standard Method A and B), respectively. When the test bar deflected by 341 μm, the temperature of the medium was recorded. Two sample bars of all PHB-PLA-plasticizer and PHB-PLA reference samples were subjected to measurements simultaneously for a given load.

### 2.10. Migration Tests—Plasticizers Migration from PHB-PLA Blends

Migration tests of PHB-PLA-plasticizer samples were performed in a drying oven heated to 110 °C. The time of exposition to the higher temperature varied from 1 h up to 16.7 days. Details of measurement conditions and preparation of samples for testing are described in our previous work. Please see [30].

### 2.11. Tensile Tests—Mechanical Characterization

The mechanical properties of PHB-PLA-plasticizer samples (as well as reference), especially Young’s modulus, the elongation at break, and the tensile strength, were determined using the device and according to measurement conditions described in our previous work. Please see [30]. The samples in the form of filaments and 3D printed double-paddle testing samples (dogbones, ~(4 × 2 × 75) mm were measured. The test approached the CSN EN ISO 527-1 standard [51]. For each sample, at least twelve tensile test measurements were carried out.

### 2.12. Charpy and Tensile-Impact Strength Tests—Mechanical Characterization

Charpy V-notched as well as Charpy un-notched tests were performed using Charpy pendulum Zwick 5113.100 (ZwickRoell GmbH & Co., Ulm, Germany) according to CSN EN ISO 179-1/eA and ISO 179-1/eU standard specifications at ambient conditions [52]. The sample bars (80 × 10 × 4) mm were 3D printed from all PHB-PLA-plasticizer filaments (3D model was generated simply by only perimeters) and half of them were notched (notch Type A). 2.7 J and 10.8 J hammers were used for the measurements of impact energy. Ten V-notched and 10 un-notched 3D printed PHB-PLA-plasticizer specimens were always measured in the edgewise position. The measurements were carried out 7 days after 3D printing. The Charpy un-notched and notched impact strengths, *a_cU_*, and *a_cN_* (kJ/m^2^), were calculated from measured and corrected impact energies *E_c_* (J). Because the breakage did not occur in the case of some flexible PHB-PLA-plasticizer specimens, we decided to also add the determination of tensile-impact strength.

The tensile-impact strength tests were performed using a Charpy pendulum impact hammer (type BPI-25COMC.10, ZwickRoell GmbH & Co., Ulm, Germany), according to CSN EN ISO 8256 standard specifications at ambient conditions [53]. The sample bars (80 × 10 × 4) mm were 3D printed from all PHB-PLA-plasticizer filaments (3D model was generated simply by only perimeters) and were notched according to the standard (type 1 notches, type 1 test specimens). A 7.5 J pendulum hammer was used with the deflection angle of 147°, and the tests were performed according to method A. The measurements were carried out 7 days after 3D printing. The notched tensile-impact strengths, *a_tN_* (kJ/m^2^), of all PHB-PLA-plasticizer specimens were calculated from measured and corrected tensile-impact energies, *E_c_* (J).

### 2.13. Optical microscopy—Structural Characterization

The sample bars (80 × 10 × 4) mm were 3D printed from all PHB-PLA-plasticizer filaments (3D model was generated simply by only perimeters). Three-dimensional printed samples were kept in the refrigerator for 30 min and then disrupted via grip and hammerlock to achieve a brittle fracture. The brittle fractures of all PHB-PLA-plasticizer samples were observed using an Olympus SZ51 optical microscope (Olympus Europa Holding GmbH, Hamburg, Germany), with the ocular WHSZ10x-H/22, 4x zoom magnification, 40 total magnifications, and transmitted LED illumination.

## 3. Results

### 3.1. Plasticizers Miscibility with PHB-PLA

Acetyltris(2-ethylhexyl)citrate (A2-EH), poly(ethylene glycol)bis(2-ethylhexanoate) (PEG-2-EH), and tris(2-ethylhexyl)citrate (C2-EH) plasticizers were kneaded with PHB-PLA mixture in the weight ratio (wt.%) 60PHB-25PLA-15 plasticizer.

**Figure 4:**
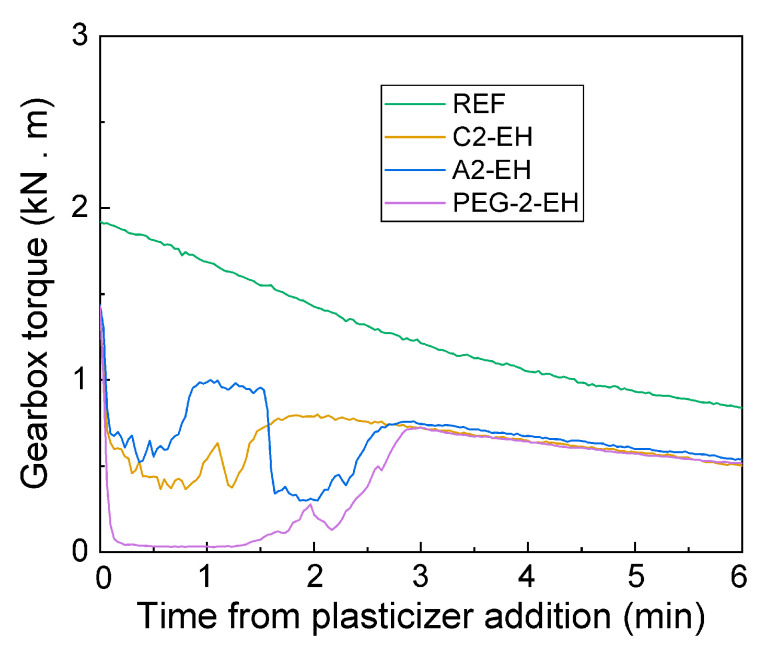
Dependence of gearbox torque on the time elapsed from the addition of plasticizers to the poly(3-hydroxybutyrate) and poly(lactic acid) (PHB-PLA) mixture.

**Figure 5:**
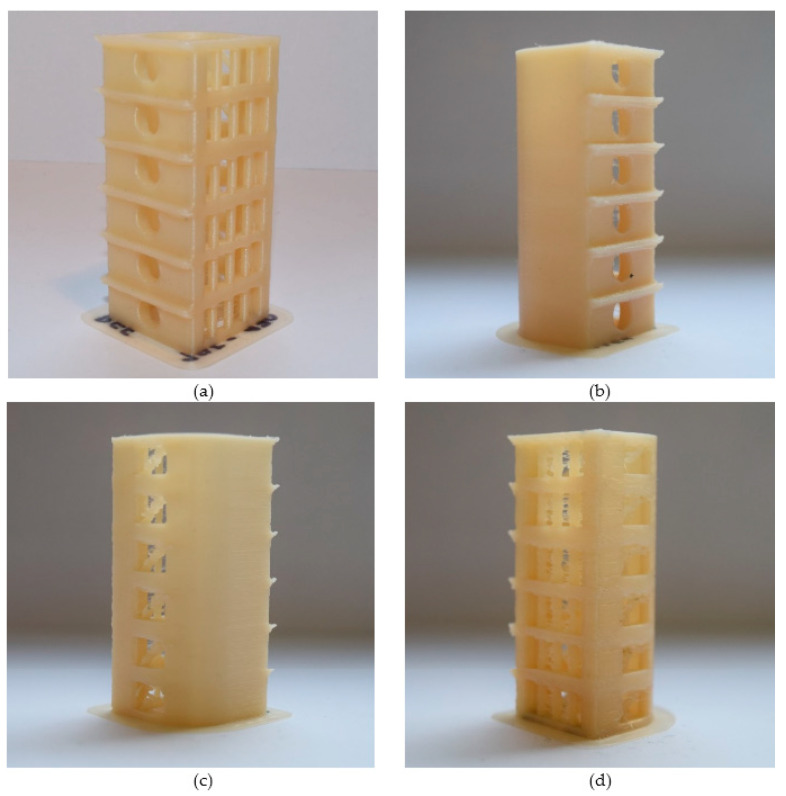
Examples of temperature towers 3D printed at 195–170 °C (minus 5 °C for each floor) from PHB-PLA-plasticizer filaments: (**a**) reference without plasticizer, (**b**) with C2-EH plasticizer, (**c**) with A2-EH, (**d**) with PEG-2-EH.

The gearbox torque dependence (kN∙m) on the time after the plasticizer was added to the PHB-PLA mixture can be seen in Figure 4. The PHB-PLA mixture had already been dosed for 1 min and kneaded for 2 min. These dependencies (of plasticized samples) are compared with the reference sample (70-30 PHB-PLA in wt.%). In the case of PEG-2-EH (see Figure 5), the gearbox torque first declined to 0 kN∙m after the plasticizer addition due to the lubrication of the blades by the plasticizer. These results are in agreement with our previous work, in the case of C-4 and A-4 plasticizers [30]. The torque resistance approached zero because the blades could move freely. After, the gearbox torque increased, because the plasticizer started to mix with PHB-PLA. Finally, the gearbox torque achieved the maximum, which corresponds to the moment of the plasticizer blending with the PHB-PLA. All plasticizers (in the concentration of 15%) were miscible with PHB-PLA within maximally 6 min of total kneading time, which corresponds to a maximum of 3 min from when the plasticizers were added. The addition of C2-EH and A2-EH plasticizers did not cause the gearbox torque declination to 0 kN∙m, which may mean a smaller lubrication effect of these plasticizers. The plasticizer C2-EH were kneaded into the PHB-PLA mixture faster than the plasticizers A2-EH and PEG-2-EH. The plasticizer with the acetyl group in the structure (A2-EH) needs more kneading time. This result is in good agreement with our previous work [30], where the plasticizer with the acetyl group A-4 (acetyl tributyl citrate) needed more kneading time compared to the plasticizer C-4 (tributyl citrate). The plasticizer structure also became evident for PEG-2-EH, where the kneading time was the longest due to a longer polymeric chain. As can be seen, all selected plasticizers were successfully kneaded with the PHB-PLA mixture.

### 3.2. 3D Printing and Printability Tests

Printability tests, specifically the temperature tower, warping test, and Benchy test, were carried out for the optimization of printing conditions, especially of printing temperature.

#### 3.2.1. Temperature Tower

The range of applicable printing temperatures was gathered via the temperature tower test described in Materials and Methods (Section 2.5.1.).

The temperature of the last floor of each temperature tower, which was successfully and completely printed (before the next floor printing failed due to too low temperature), can be seen in Table 3. In the case of higher temperatures (temperature towers in the range 220–195 °C), all floors were successfully printed. Contrariwise, the material degradation due to higher temperature can be seen as darker floor coloration, and thus, higher temperatures were not selected into the range of applicable printing temperatures. Equally, the printing with low temperatures is not well reproducible (due to the fluctuation of the nozzle temperature and other printing conditions), although geometric elements are very well printed at lower temperatures. Moreover, the layers do not hold together so well, and sometimes, delamination can occur during printing at lower temperatures. The evaluated range of applicable printing temperatures are given in Table 3. As can be seen, the range of applicable temperatures for all PHB-PLA-plasticizer filaments was 160–195 °C. In contrast, the filament with a non-plasticized reference has the largest range of applicable temperatures (160–200 °C). Subjective evaluation (marks from 1–5, where 1 is the best) of stringing, some geometric elements (especially colonnade and diagonal crossbar in a rectangle), and undesirable holes in the 3D printed structure can be seen in Table 4. This table contains only a few elements as an example of possible evaluation, although all geometric elements of the temperature tower might be evaluated (as was described in Section 2.5.1). The average marking from this subjective evaluation for all temperature towers can also be seen in Table 4. The printability of geometric elements of the reference sample (without plasticizer) is the best and for the largest range of temperatures. In contrast, the PHB-PLA-PEG-2-EH filament had the worst printability of geometric elements. The filaments with C2-EH and A2-EH had sufficient printability of geometric elements (comparable to reference sample) but only at the lowest temperatures. It seems that it is caused by lower viscosity of their melting during printing at higher temperatures and so shapes of geometric elements collapsed more than during printing at lower temperatures.

This evaluation via the comparison of printed elements is rather subjective and requires some practice. Hence, the temperature tower gave us information only about the range of applicable temperatures. These ranges of applicable temperatures were then optimized via the warping tests to obtain one “ideal” temperature for the printing of specimens for mechanical and thermo-mechanical characterizations. These specimens must be printed reproductively and, due to their size, also with the lowest warping. Thus, despite the fact that the printability of geometric elements is best at a particular temperature, this temperature is not suitable for printing the specimens for testing. Extreme temperatures (too high or low) must be taken into consideration, and it is not suitable to include them in the applicable temperatures. This method is possible to apply more exactly using image analysis of geometric elements. However, visual evaluation was fully sufficient for the selection of the range of applicable temperatures in our case. Image analysis of geometric elements could be useful, e.g., in the case of scaffold printing and other products with a demand for high-level accuracy. The advantages of the temperature tower are the possibility to examine a wide range of materials printed within a wide range of temperatures and to debug the printing conditions on different types of printers. The examples of 3D printed temperature towers from PHB-PLA-plasticizer filaments can be seen in Figure 5.

#### 3.2.2. Warping

For each PHB-PLA-plasticizer sample, warping specimens were printed for each chosen applicable temperature. The temperature of the first layer for each specimen was set to 195 °C (according to the experience with our material [46]). The warping coefficient is a dimensionless quantity and was calculated as described in Materials and Methods (see Equation (1)). The later the negative effect of warping started to manifest, the lower the warping coefficient is and the better is the printability of a material. In Table 5 we can see the results of warping coefficient measurements. The best warping coefficient (the lowest) and appropriate printing temperature is highlighted for each PHB-PLA-plasticizer filament. As can be seen, the warping coefficient is usually lower with decreasing temperature. In the case of the non-plasticized reference, the lowest warping (1.1 ± 0.1) was achieved at 180 °C, filaments with PEG-2-EH plasticizer had the lowest warping (2.0 ± 0.2) at 180 °C. Filament with A2-EH plasticizers had comparable warping (1.8 ± 0.0)‒(2.0 ± 0.1) at all temperatures, so we decided that 185 °C would be the best temperature for the printing. The exception was in the case of filament with C2-EH plasticizer, where the warping coefficient was higher with decreasing temperature. In contrast, the lowest warping for C2-EH filament (2.3 ± 0.1) was achieved at a higher temperature of 195 °C. The reason for this effect is not clarified now.

Although the printability of geometric elements in the case of C2-EH and A2-EH plasticizer is best when printing at lower temperatures (180–155 °C), the warping seems to be better at higher temperatures.

This quick method is established on unaided vision, is subjective, and the measurements should be carried out by one person to minimalize the error rate. This method is suitable for a basic overview and comparison of printing properties between samples. In the case of more complicated and accurate samples (e.g., scaffolds), more precise measurements would be employed.

#### 3.2.3. Test Specimen Printing and Verification of Printing Conditions Using 3D Benchy

**Figure 6:**
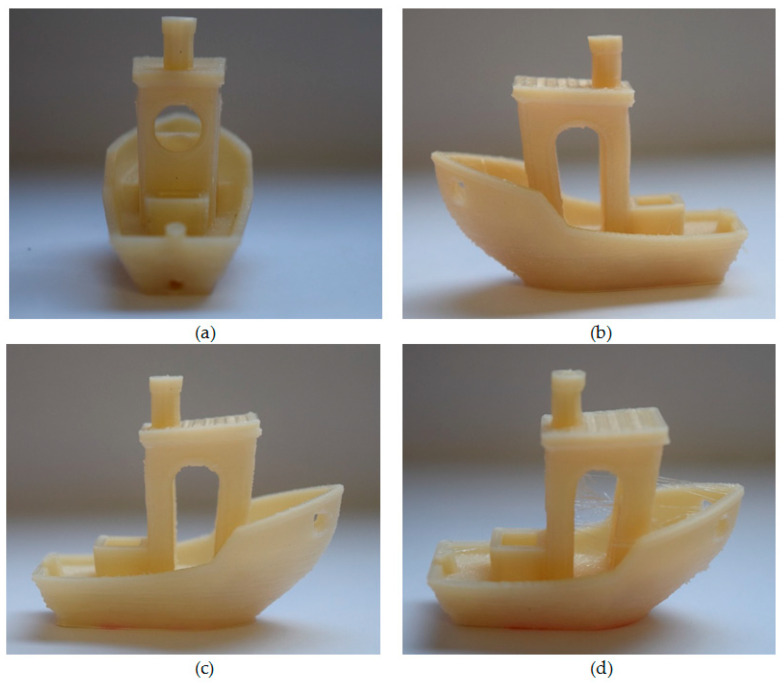
Three-dimensional Benchies printed under the conditions optimized via the temperature tower and warping tests, from all PHB-PLA-plasticizer filaments: (**a**) reference without plasticizer, (**b**) with C2-EH plasticizer, (**c**) with A2-EH, (**d**) with PEG-2-EH.

Final verification of adjusted printing conditions, especially printing temperatures, proceeded via the Benchy test. Benchies were printed at the same printing conditions as the test specimen samples (described in Table 2 and Table 5) from all PHB-PLA-plasticizer filaments. Printed 3D Benchies were then visually evaluated [47], and the printing conditions were revised. Although there are many possible signs for the Benchy evaluation, we subjectively marked only some of them 1–5 (where 1 is the best) and this way visually examined the quality of printed Benchies. We were interested in signs which were related to the material properties rather than to the adjustment of our printer. In Table 6 and Table 7, we can see some chosen Benchy signs (shape features [47] and other signs related to the material) and their subjective marking. In Table 8, the average mark of all PHB/-PLA-plasticizer Benchies calculated from this evaluation can be seen. The Benchy from the reference filament (without plasticizer) has the best printability. The Benchies printed from filaments with A2-EH and C2-EH plasticizers also had comparable printability. The printability of Benchy printed from filament with PEG-2-EH plasticizer seemed to be the worst. The examples of 3D Benchies printed from PHB-PLA-plasticizer filaments can be seen in Figure 6.

The Benchies were printed at nozzle temperatures selected via the warping test. The evaluation of benchies confirmed these temperatures (see Table 5) as the most suitable for the printing of testing specimens.

### 3.3. Modulated Differential Scanning Calorimetry—Thermal Characterization

**Figure 7:**
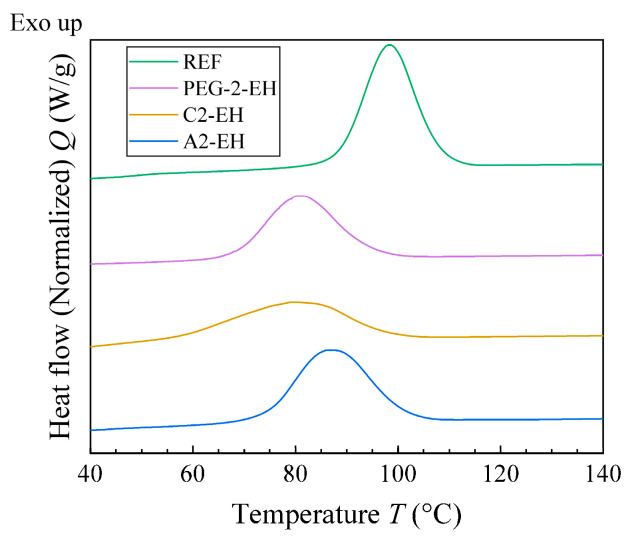
First cooling scan (differential scanning calorimetry (DSC)) of the non-plasticized reference sample (REF) and PHB-PLA-plasticizer blends (the plasticizers are PEG-2-EH, C2-EH, and A2-EH).

**Figure 8:**
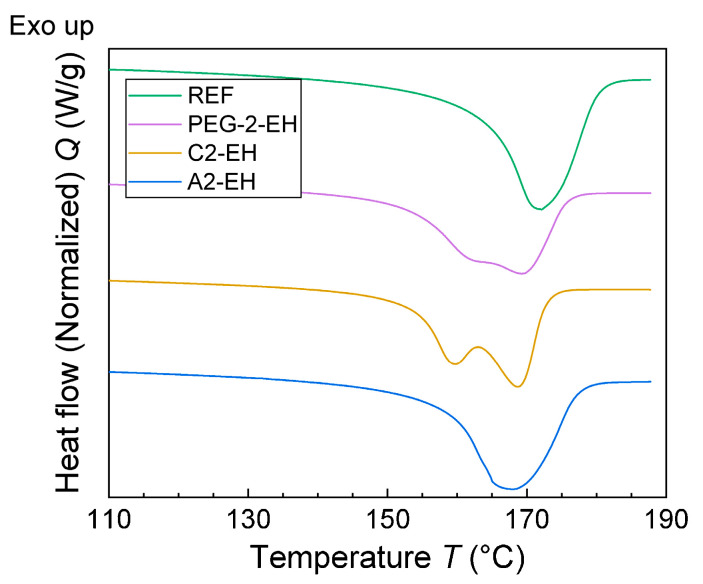
Second heating scan (DSC) of the non-plasticized reference sample (REF) and PHB-PLA-plasticizer blends (the plasticizers are PEG-2-EH, C2-EH, and A2-EH).

**Figure 9:**
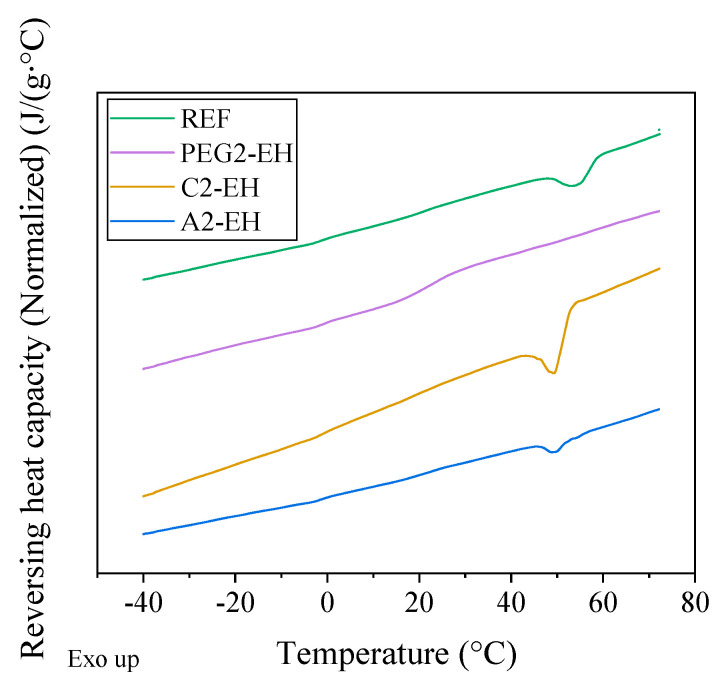
Reversing heat capacity (from modulated differential scanning calorimetry (MDSC) scan) of the non-plasticized reference sample (REF) and PHB-PLA-plasticizer blends (the plasticizers are PEG-2-EH, C2-EH, and A2-EH).

The combination of modulated and conventional differential scanning calorimetry (MDSC and DSC, respectively) was used for observation of the thermal behavior of PHB-PLA-plasticizer samples and a non-plasticized sample (reference). The glass transition temperature of PHB could not be detected due to the high amount of rigid fraction in PHB. With the help of the melting and crystallization temperatures shifts and using the crystallinity change, the effect of the plasticizers on the thermal properties of PHB could be observed (Figure 7 and Figure 8, respectively). In Figure 9 we can see the change in *T*_g_ in the case of PLA fraction in blends. Measured and evaluated data are presented in Table 9.

Low molecular plasticizer introduced into the PHB-PLA blend should cause greater mobility of macromolecular chains. Consequently, the melting and the crystallization temperatures should be shifted to lower temperatures. The melting temperature, *T*_m_, of PHB-PLA-plasticizer blends was not significantly shifted as compared to the non-plasticized reference. C2-EH, PEG-2-EH, and A2-EH plasticizers shifted especially for the crystallization temperature, where *T*_c_ was lower by 19 °C in the case of C2-EH (as compared to the reference), by 17 °C in the case of PEG-2-EH, and by 12 °C in the case of A2-EH. Low molecular plasticizers are retained in the amorphous phase in between crystallites in the case of a semicrystalline polymer. In the case of our blend of PHB semicrystalline and PLA amorphous polymer, we assumed that additives (plasticizers) are ejected into the amorphous polymer during crystallization. Thus, the plasticizing effect is more evident when we are monitoring the change of PLA *T*_g_. PLA *T*_g_ in the case of esters of citric acid decreased only slightly by 6.2 °C for C2-EH and only by 4.1 °C for A2-EH. Thus, in the case of C2-EH and A2-EH plasticizers, increased length of alkyl chains (compared to tributyl citrate and acetyl tributyl citrate from our previous work [30]) caused a worse plasticizing effect and led to *T*_g_ being higher than 52 °C. A2-EH plasticizer exhibits the highest PLA *T*_g_ and *T*_c_ temperatures of all plasticized samples and seems to have the worst plasticizing ability. Both blends, with A2-EH and C2-EH, are expected to be glassy at laboratory temperature. PEG-2-EH plasticizer with its long but not-branched structure, which seems to have a great plasticizing effect, caused the decrease of PLA *T*_g_ by almost 37 °C and the decrease of *T*_c_ by 17 °C, as compared to the reference. On the contrary, the melting temperature, *T*_m_, of blends with PEG-2-EH plasticizer decreased by only 3 °C.

The crystallinity of PHB (*X*_c_ in%) in the reference and plasticized blends was calculated from the melting enthalpy (*H*_m_ (J/g)) measured during the second heating DSC scan (Table 9). The non-plasticized reference had the crystallinity of PHB 63%. Samples plasticized using PEG-2-EH and C2-EH had lower crystallinity of PHB (about 55%). The plasticizer A2-EH had even higher crystallinity (66%) than the reference. This could explain the worst plasticizing effect of A2-EH.

### 3.4. Oscillation Rheometry—Thermal Characterization

**Figure 10:**
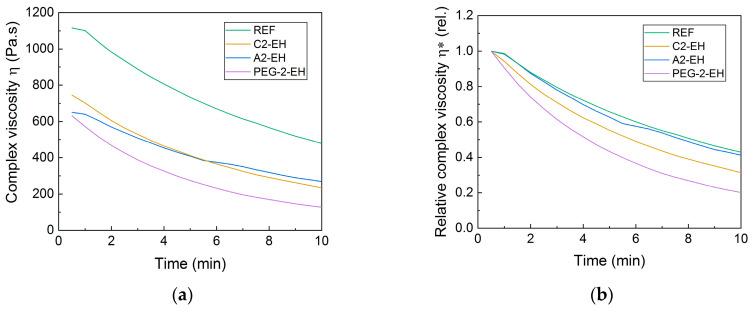
Complex viscosity (**a**) and relative complex viscosity (**b**) evaluated from oscillation rheometry (RPA) measurements of the non-plasticized reference sample (REF) and PHB-PLA-plasticizer blends (where the plasticizers are PEG-2-EH, C2-EH, and A2-EH). Measured 7 days after the preparation of blends.

The oscillation rheometry (RPA measurements) was carried out 7 and 40 days after the blend preparation. Complex viscosity (Pa∙s) of all PHB-PLA-plasticizer samples measured after 7 days from the preparation of pellets can be seen in Figure 10. For better comparison, the relative complex viscosity for all samples was evaluated as well. All plasticized samples had apparently lower complex viscosity during the test compared to the reference. The blends with PEG-2-EH plasticizer had the lowest complex viscosity at the beginning of the measurement and the largest decrease of complex viscosity, similarly to the blend with C2-EH plasticizer. The blend with PEG-2-EH plasticizer showed the most significant degradation of the polymeric matrix among all plasticized blends. It correlates well with subjective printability results, where the plasticizers C2-EH and PEG-2-EH had the worst printability.

**Figure 11:**
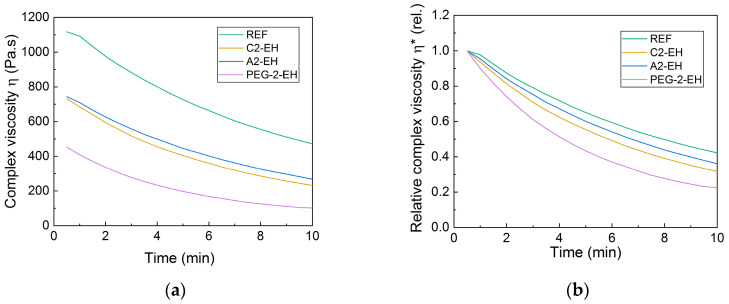
Complex viscosity (**a**) and relative complex viscosity (**b**) evaluated from RPA measurements of the non-plasticized reference sample (REF) and PHB-PLA-plasticizer blends (where the plasticizers are PEG-2-EH, C2-EH, and A2-EH). Measured 40 days after the preparation of blends.

The complex viscosity (Pa∙s) of all PHB-PLA-plasticizer samples measured after 40 days from the preparation of pellets can be seen in Figure 11. While the blends with A2-EH plasticizer seem to have a little higher complex viscosity at the beginning of the measurement (after 40 days of aging), in contrast, the blends with PEG-2-EH plasticizer had a little lower complex viscosity at the beginning of the measurement, compared to the measurements carried out after 7 days. Complex viscosities of the reference and the sample with C2-EH measured after 40 days correspond to the measurements after 7 days from the preparation of pellets. At the end of the measurement after 40 days, the blends with PEG-2-EH plasticizer had lower complex viscosity. The relative complex viscosity can be also seen in Figure 11. The shapes of relative complex viscosity curves of blends with A2-EH plasticizer seem to be shifted from the reference down towards C2-EH, compared to the measurements after 7 days. The blends with PEG-2-EH plasticizer had the lowest relative complex viscosity.

In order to detect what happened to the blend with PEG-2-EH plasticizer and to confirm its degradation, GPC (gel permeation chromatography) measurements were additionally performed. 

**Figure 12:**
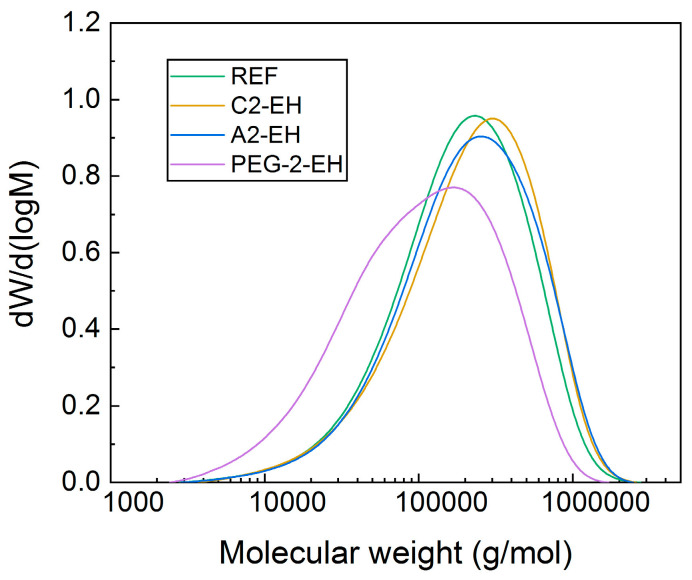
Gel permeation chromatography (GPC) measurements of the non-plasticized reference sample (REF) and PHB-PLA-plasticizer blends (where the plasticizers are PEG-2-EH, C2-EH, and A2-EH).

The differential distribution of molecular weights for all PHB-PLA-plasticizer samples and the reference can be seen in Figure 12. The curves correspond to the PHB-PLA distribution of molecular weights, and as can be seen, the reference and other plasticized samples except for the sample with PEG-2-EH plasticizer had a similar distribution of molecular weights. Plasticized samples (except for PEG-2-EH) even caused the shift to larger molecular weights, compared to the reference. The distribution of molecular weights of the PHB-PLA-PEG-2-EH sample is significantly shifted to lower molecular weights, so PHB-PLA from this sample is evidently degraded during the processing, which confirms the RPA results. Figure 12 displays only the principal peak of polymer elution. Plasticizers are not displayed in this graph, but they created an individual peak (under 1000 g/mol). Regarding high measurement error in this area (on the periphery of the range of our GPC column), these peaks are not displayed. The influence of contained plasticizer on the properties of solvent (or GPC analysis result) is theoretically possible, but we do not think this influence is radical in practice. Sample concentration for GPC analysis was 0.5% and during elution was further diluted below 0.005%. The measurements were carried out identically and with a similar error, so we think it justifies this kind of comparison.

### 3.5. HDT—Heat Deflection Temperature—Thermal Characterization

**Figure 13:**
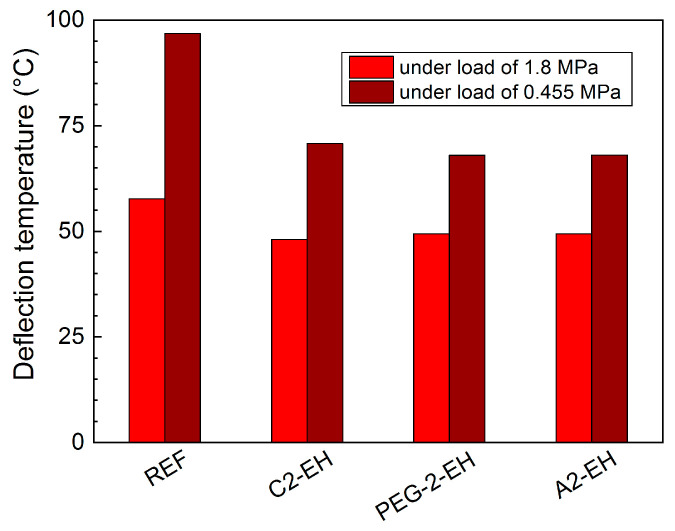
Heat distortion temperature (HDT) measurements of the non-plasticized reference sample (REF) and PHB-PLA-plasticizer blends (where the plasticizers are PEG-2-EH, C2-EH, and A2-EH).

In Figure 13, you can see the HDT for all the PHB-PLA-plasticizer samples and for the reference. As you can see, the plasticizing effect manifests itself by decreasing the HDT under both loads, compared to the reference. The HDTs of the plasticized samples measured under a higher load of 1.8 MPa were decreased only slightly, compared to the reference, specifically, in decreasing order from the reference (HDT = 57.7 °C): A2-EH, PEG-2-EH, C2-EH (HDT = 48.1 °C). The differences in the HDT are slightly more apparent for the measurements under a lower load of 0.455 MPa. The HDT decreased: from reference (96.8 °C), through the blends with plasticizers C2-EH (70.8 °C), A2-EH to PEG-2-EH (67.9 °C). The differences between the measured HDTs of the plasticized samples are relatively small.

### 3.6. Migration Tests—Plasticizers Migration from PHB-PLA Blends

The tests of plasticizer migration from samples were performed at 110 °C in a drying oven (as was described in Materials and Methods). We observed plasticizer weight loss (%) after, up to 16.7 days.

**Figure 14:**
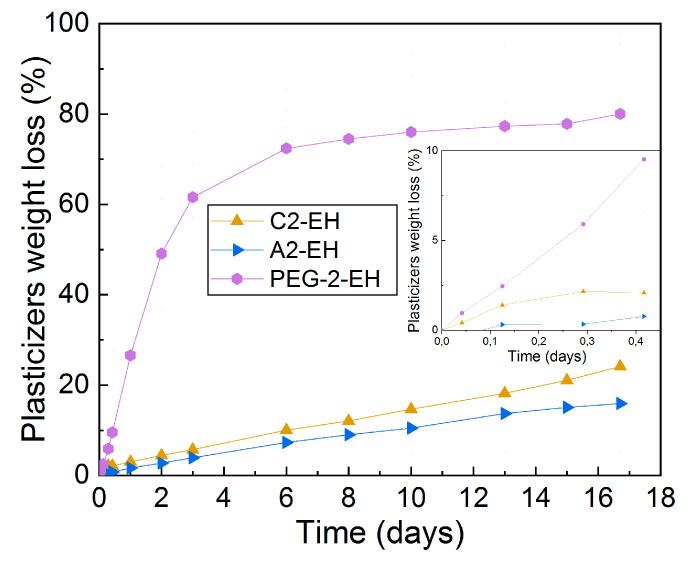
Weight loss of plasticizers (%) from PHB-PLA-plasticizer blends after up to 16.7 days of exposition to 110 °C in the drying oven.

The weight loss (%) of C2-EH, A2-EH, and PEG-2-EH plasticizers from samples after 1, 3, 7, 10 h, and 1, 2, 3, 6, 8, 10, 13, 15, and 16.7 days of exposition can be seen in Figure 14.

The plasticizer PEG-2-EH started to migrate very fast after 10 h of exposition. The PEG-2-EH plasticizer showed up to 80.0% weight loss at the end of the test. In contrast, the plasticizers which showed the best resistivity to diffusion were C2-EH and A2-EH. Their weight loss proceeded gradually and showed only 24% and 16% weight loss at the end of the test, respectively.

Our previous study [30] showed the rapid migration of tributyl citrate and acetyl tributyl citrate plasticizers. These plasticizers had smaller molecular weight and shorter lengths of alkyl chains linked to citrate molecules. On the other hand, C2-EH and A2-EH plasticizers have longer side alkyl chains, are more branched, and have a greater molecular weight. Thus, C2-EH and A2-EH plasticizers demonstrated the best migration stability and did not diffuse from the sample so much.

PEG-2-EH plasticizer has a greater molecular weight but has a linear non-branched structure and migrates also very fast. The GPC (gel permeation chromatography) confirmed its degradation effect on PHB-PLA. This influence of structure on diffusion is in good agreement with [29,30,31,32,33,34].

The weight loss (%) measurements were also carried out on the reference sample. An insignificant decrease in the reference weight loss was measured, which we attribute to water evaporation (0.3% after 1 h, 0.4% after 2–16.7 days). These continuous values were subtracted from other samples with plasticizers.

### 3.7. Tensile Tests—Mechanical Characterization

The tensile tests were performed on PHB-PLA-plasticizer filaments after 7 and 40 days from the extrusion and after three days of exposition to 110 °C in the drying oven and on 3D printed dogbones after 7 days from printing.

**Figure 15:**
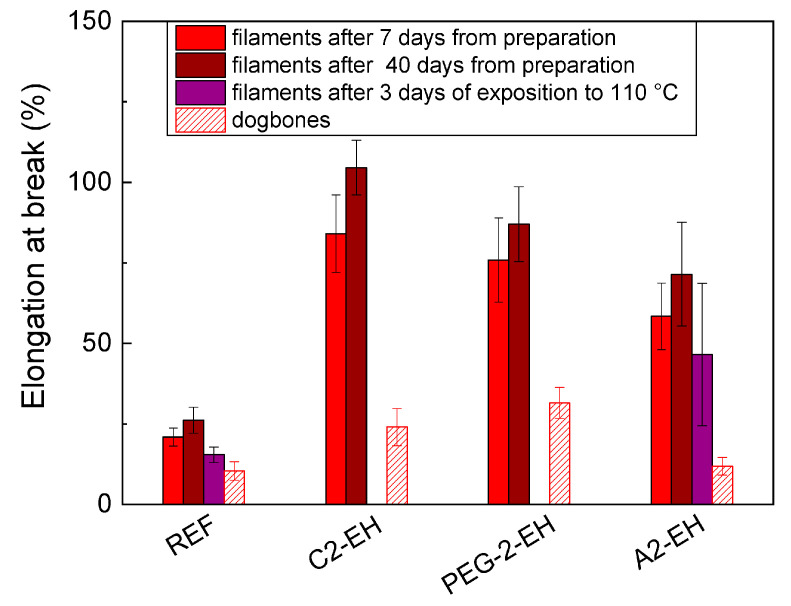
Elongation at break (%) of neat PHB-PLA (reference), PHB-PLA-plasticizers filaments, and 3D printed dogbones.

The elongation at break is for us the most interesting quality considering plasticizer efficiency. In Figure 15, we can see the measured elongation at break (%) of all PHB-PLA-plasticizer samples compared with the non-plasticized reference. The plasticizers’ influence can be clearly seen, and all three plasticizers have a positive softening effect in PHB-PLA polymers.

The plasticizers A2-EH, C2-EH, and PEG-2-EH improved the elongation at break (measured after seven days from the filament preparation) rather slightly: from 21% for non-plasticized PHB-PLA filament samples to 58% (A2-EH), 84% (C2-EH), and 76% (PEG-2-EH) for plasticized filaments. The plasticizing effect of these plasticizers is not so significant; on the other hand, they showed better resistivity to diffusion (except PEG-2-EH, as was proved in the migration tests Section 3.6). Their elongation at break measured after 40 days is even slightly higher than that after 7 days, as well as the elongation at break of the non-plasticized reference measured after 40 days. We assumed that this is within the frame of measurement error. Nevertheless, the blends with A2-EH and C2-EH plasticizers are relatively resistant to aging. The plasticizer structure apparently influenced the results. Their weaker softening effect was influenced by their more branched structure and increased length of alkyl chains linked to the citrate molecule. On the other hand, they have stronger resistivity to aging and higher temperatures. The MDSC measurements correspond with tensile test results, where A2-EH and C2-EH plasticizers showed smaller *T*_g_ reduction and thus worse plasticizing effect. The PEG-2-EH plasticized sample seems to be an exception; according to the migration tests, this plasticizer is not resistant to diffusion at higher temperatures. However, it seems to be resistant to aging. The elongation at break of the blends with A2-EH plasticizer decreased only slightly after three days of exposition to 110 °C. On the contrary, the filaments with C2-EH and PEG-2-EH plasticizers after three days of exposition to 110 °C were so fragile that the elongation at break could not have been measured. This is the consequence of PEG-2-EH sensitivity to higher temperatures, confirmed by the RPA measurements. The elongation at break of dogbones with A2-EH, C2-EH, and PEG-2-EH plasticizers confirmed the weaker plasticizing effect.

Tensile test measurements of filaments with A-4 and C-4 plasticizers have already been published in our previous work [30], and these results can serve as a comparison with other plasticizers, to see how plasticizer structure influences elongation at break.

In Table 10 and Table 11 we can see Young’s modulus (GPa) and the tensile strength (MPa) of the PHB-PLA-plasticizer samples and non-plasticized reference. The weaker softening effect of plasticizers can be seen also from these results. The tensile strength values of the plasticized samples were comparable for all the plasticizers except for PEG-2-EH, which showed lower tensile strength measured 7 days after preparation. The samples with A2-EH plasticizer had comparable values of tensile strength not only 7 and 40 days after preparation, but also after 3 days at 110 °C.

Armentato et al. [38] investigated PLA-PHB-lactic acid oligomers (OLA) blends and achieved significant improvement of ductile properties by the introduction of OLA. OLA caused a meaningful decrease of the tensile strength, and the elongation at break increased with the increasing of OLA concentration. They achieved an improvement of elongation at break from 140% for non-plasticized 85PLA-15PHB blends to 370% for a 55PLA-15PHB-30OLA blend.

Arrieta et al. [26] observed decreased tensile strength and modulus and increased elongation at break with A4 and PEG plasticizers. Blends plasticized with A4 had higher thermal stability and flexibility than blends plasticized with PEG. They achieved an improvement of elongation at break from 2.0% for the PLA-PHB blend to 182% for the PLA-PHB-A4 blends. Ductile properties of PLA-PHB blends were not clearly improved by the addition of PEG plasticizer.

Abdelwahab et al. [3] investigated the PLA-PHB-Lapol system. The addition of Lapol into the PLA-PHB system caused a decrease of Young’s modulus and the tensile strength and a slight increase of elongation at break from 7% to 15%.

### 3.8. Charpy and Tensile-Impact Strength Tests—Mechanical Characterization

Twenty sample bars (80 × 10 × 4) mm were 3D printed from all PHB-PLA-plasticizer filaments (3D model was generated simply by only perimeters), half of them were notched (V-notch Type A) and half of them were left un-notched. The measurements were carried out 7 days after 3D printing. The Charpy un-notched and notched impact strengths, *a_cU_*, and *a_cN_* (kJ/m^2^), were calculated from measured and corrected impact energies, *E_c_* (J). Because un-notched breakage did not occur in the case of too flexible PHB-PLA-PEG-2-EH specimens (even with a 10.8 J hammer), we decided to supplement the tests also using the determination of tensile-impact strength. V-notched breakage occurred in the case of all PHB-PLA-plasticizer specimens with a 2.7 J hammer, except for the specimen with PEG-2-EH plasticizer, where the 10.8 J hammer was necessary to break the specimen.

The tensile-impact strength measurements were carried out 7 days after 3D printing. The notched tensile-impact strengths, *a_tN_* (kJ/m^2^), of all PHB-PLA-plasticizer specimens were calculated from measured and corrected tensile-impact energies, *E_c_* (J).

**Figure 16:**
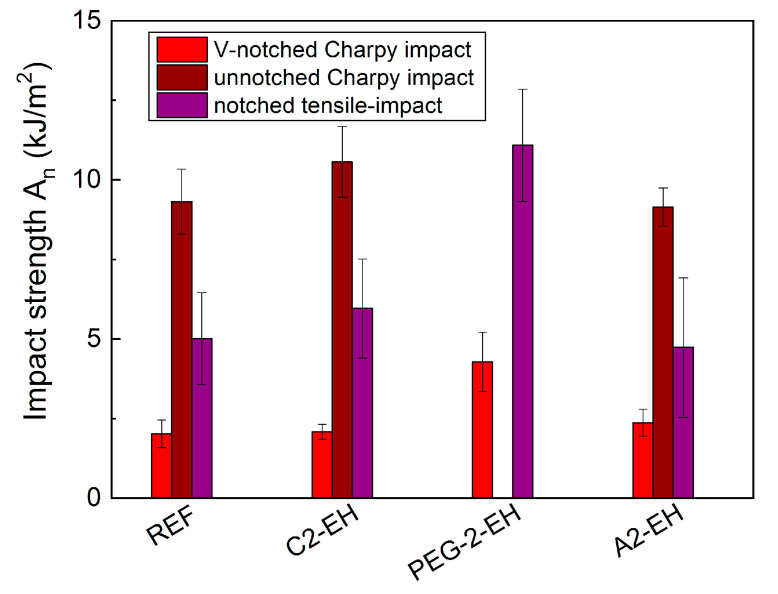
Charpy and tensile-impact measurement-evaluated impact strength of neat PHB-PLA (reference) and PHB-PLA-plasticizer filaments.

As can be seen in Figure 16, PHB-PLA samples with C2-EH and A2-EH plasticizers almost did not influence evaluated Charpy and tensile-impact strength, *A*_n_ (kJ/m^2^), as compared to the non-plasticized reference. In the case of a sample with A2-EH plasticizer, un-notched Charpy and notched tensile-impact strength are even slightly lower. Their evaluated impact strength is within the error frame, especially the tensile-impact measurements had quite a large error frame. The evaluated impact strength was not influenced only by the material properties, but also by the structure of the 3D printed specimens. The breakage in the case of tensile-impact tests proceeded along 3D printed perimeters. The enhancement of impact strength measured from V-notched Charpy and notched tensile-impact is apparent for the samples with PEG-2-EH plasticizers. Notched tensile-impact strength of PHB-PLA-PEG-2-EH samples only slightly increased to 11 kJ/m^2^, as compared to 5 kJ/m^2^ of the non-plasticized reference.

### 3.9. Optical Microscopy—Structural Characterization

**Figure 17:**
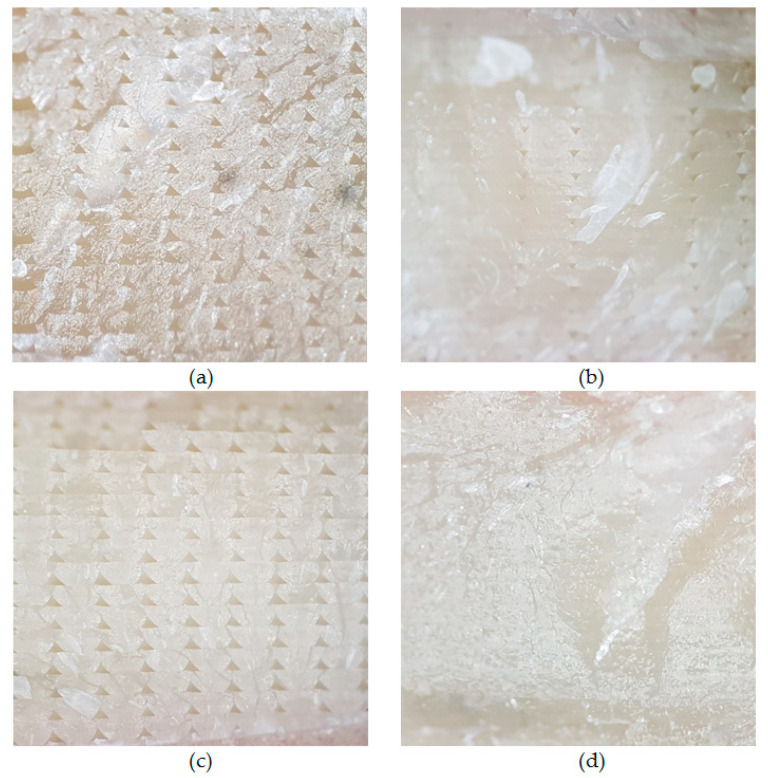
The surface of 3D printed samples using optical microscopy: (**a**) reference without plasticizer, (**b**) with C2-EH plasticizer, (**c**) with A2-EH, (**d**) with PEG-2-EH.

The brittle failures of all PHB-PLA-plasticizer samples were optically analyzed using Olympus SZ51 an optical microscope (see Materials and Methods). The samples were 3D printed under the conditions optimized via the temperature tower and warping tests, equal to conditions which were applied also to the printing of testing specimens for mechanical and thermal characterizations. This elemental verification of 3D printed structure via optical microscopy is a cheap and easy way to check the printing settings and conditions. In Figure 17, we can see that the structures of all PHB-PLA-plasticizer samples are quite compact and free of large holes and missing filaments, which means this is quite a good setting of printing conditions. The biggest holes and the potential for upgrading the printing conditions (especially printing temperature) can be seen on the reference and A2-EH samples. Generally, the printing conditions seem to be adjusted well.

## 4. Conclusions

Poly(3-hydroxybutyrate)-poly(l,d-lactic acid)-plasticizer blends in the weight ratio (wt.%) of 60-25-15 were successfully prepared using a corotating twin-screw extruder and then using a single screw extruder to achieve filaments with defined diameters for further 3D printing via the fused deposition modeling method. Three low molecular plasticizers were used. Two of them were based on esters of citric acid (acetyl tris(2-ethylhexyl) citrate (A2-EH) and tris(2-ethylhexyl)citrate (C2-EH)), and the last one is poly(ethylene glycol) bis(2-ethylhexanoate) (PEG-2-EH). All plasticizers were well miscible with the PHB-PLA matrix (with PHB majority). The printability of 3D samples printed from these blends was judged according to newly designed tests—the “Temperature tower” and the “Warping specimen”. The influence of particular plasticizers on the printability of geometrical shapes and on warping was investigated. Due to these tests, we revealed the best nozzle printing temperature for each PHB-PLA-plasticizer blend, which varied in the range of 180‒195 °C. After this optimization, we were able to print the testing samples with the best printing quality and with minimal warping for further mechanical, thermal, and optical measurements. The printing quality of the test specimens was then checked using an optical microscope and the 3DBenchy test. The 3DBenchies from all PHB-PLA-plasticizer filaments were successfully printed and visually marked. The used plasticizers seemed to have a quite similar influence on the “printability”, except for PEG-2-EH, with which the worst Benchy was printed.

The softening effect of plasticizers was not so significant. They improved the elongation at break rather slightly: from 21% for non-plasticized PHB-PLA filament samples to 58% in the case of A2-EH, 84% in the case of C2-EH, and 76% in the case of PEG-2-EH. In spite of the fact that the values of measured elongation at break of 3D printed dogbones were lower than those of the samples measured in the filament form, dogbone tensile tests measurements also confirmed a less meaningful but sufficient plasticizing effect.

The minor softening effect of plasticizers was confirmed using MDSC. Still, the introduction of low molecular plasticizer into the PHB-PLA blend caused greater mobility of macromolecular chains. Consequently, the melting temperatures and the crystallization were slightly shifted to lower values. The plasticizing effect is more evident when observing the change of PLA *T*_g_ because low molecular additives are retained in the amorphous phase in between crystallites in the case of a semicrystalline polymer. C2-EH and A2-EH plasticizers showed a slight plasticizing effect, where the PLA *T*_g_ was decreased only by 6 °C (C2-EH) and by 4 °C (A2-EH). Surprisingly, the plasticizer PEG-2-EH caused a decrease of PLA *T*_g_ by 37 °C. Heat distortion temperature and the viscosity measurements using oscillation rheometry were executed as well. Gel permeation chromatography measurements were additionally performed to detect what happened to the PHB-PLA blend with PEG-2-EH plasticizer and confirmed the degradation of the polymer matrix due to the presence of PEG-2-EH.

Migration tests were performed in order to investigate the migration of all three plasticizers from the PHB-PLA matrix. The plasticizer PEG-2-EH showed quite large migration after 16.7 days of exposition to 110 °C in the drying oven. The influence of plasticizer structure on the migration process was well noticeable; esters of citric acid with larger molecular weight (C2-EH and A2-EH) showed the best stability and tendency not to migrate from the sample.

Charpy and tensile-impact tests were also carried out. The PHB-PLA samples with C2-EH and A2-EH plasticizers had negligible influence on evaluated Charpy and tensile-impact strength, *A*_n_ (kJ/m^2^), as compared to the non-plasticized reference. The improvement of impact strength measured from V-notched Charpy and notched tensile-impact is slightly apparent for the samples with PEG-2-EH plasticizer. The notched tensile-impact strength of the PHB-PLA-PEG-2-EH sample increased to 11 kJ/m^2^, as compared to 5 kJ/m^2^ of the non-plasticized reference. The evaluated impact strength was not influenced only by the material properties, but also by the structure of the 3D printed specimens. The breakage in the case of tensile-impact tests proceeded along 3D printed perimeters.

The goal was to create a new type of PHB-PLA-plasticizer blends, to sufficiently improve poor mechanical properties of neat PHB-PLA blends (with a majority of PHB) via the addition of appropriate plasticizer and mainly to investigate the printability of these blends. All three examined plasticizers are applicable and also suitable for 3D printing technology and will be later investigated especially in biomedicine as scaffolds.

## Figures and Tables

**Table 1:** Commercial and chemical names of used plasticizers and their molecular weights and labels.

Commercial	Chemical Name	Molecular Weight (g∙mol^−1^)	Plasticizer’s Label
Citrofol^®^ AHII	acetyl tris(2-ethylhexyl) citrate	570	A2-EH
poly(ethylene glycol) bis(2-ethylhexanoate)		650	PEG-2-EH
**Synthetized**	**Chemical Name**	**Molecular Weight (g∙mol^−1^)**	**Plasticizer’s Label**
**-**	tris(2-ethylhexyl) citrate	528	C2-EH

**Table 2:** Basic printing parameters.

Basic Printing Parameters	Dogbone	Temperature Tower, Warping, 3DBenchy, Other Testing Samples
Filament diameter (mm)	1.75 ± 0.05	1.75 ± 0.05
Nozzle diameter (mm)	0.4	0.4
Layer height (mm)	0.2	0.2
Perimeters printing speed (mm∙s^−1^)	40	45
Infill printing speed (mm∙s^−1^)	60	200
Bed heating (°C)	20	20
Fan (%)	100	100

**Table 3:** Evaluation of 3D printed temperature towers.

PHB-PLA-Plasticizer Filament	Temp. Range (°C)	Last Printed Floor (°C)	Range of Applicable Temperatures (°C)
REF (PHB-PLA)	220–195	220	200–195
	195–170	170	195–170
	180–155	160	180–160
C2-EH	220–195	220	-
	195–170	170	190–180
	180–155	160	180–160
A2-EH	220–195	220	-
	195–170	170	180–175
	180–155	155	180–165
PEG-2-EH	220–195	220	-
	195–170	170	195, 180
	180–155	155	165

**Table 4:** Visual evaluation of the geometric elements of some of the 3D printed temperature towers via marks 1–5 (1 is the best).

PHB-PLA-Plasticizer Filament	Temp. Range (°C)	Stringing	Colonnade	Diagonal	Holes in Structure	Average Mark
REF (PHB-PLA)	220–195	4	3	3	2	3
	195–170	2	1	1	3	1.75
	180–155	1	2	1	3	1.75
C2-EH	220–195	5	5	5	1	4
	195–170	4	4	5	3	4
	180–155	2	2	3	3	2.5
A2-EH	220–195	5	4	5	1	3.75
	195–170	4	4	5	1	3.5
	180–155	2	1	2	3	2
PEG-2-EH	220–195	5	5	5	4	4.75
	195–170	5	5	5	3	4.5
	180–155	4	3	4	2	3.25

**Table 5:** Warping coefficient calculated from warping specimen measurements carried out at some applicable temperatures (which were selected from the temperature tower results).

PHB-PLA-Plasticizer	Printing Temperature (°C)	Warping Coefficient	Selected Temperature for Specimen Printing (°C)
REF (PHB-PLA)	205	2.1 ± 0.1	180
	200	1.4 ± 0.0	
	195	1.6 ± 0.0	
	190	1.6 ± 0.0	
	185	1.3 ± 0.0	
	180	1.1 ± 0.1	
C2-EH	195	2.3 ± 0.1	195
	190	2.5 ± 0.2	
	185	2.6 ± 0.2	
	180	3.6 ± 0.3	
A2-EH	190	1.8 ± 0.0	185
	185	1.8 ± 0.0	
	180	2.0 ± 0.1	
PEG-2-EH	190	2.7 ± 0.1	180
	185	3.0 ± 0.1	
	180	2.0 ± 0.2	

**Table 6:** Subjective visual evaluation of 3D Benchies: some of their chosen signs related to the material properties. Subjective marking (1–5, where 1 is the best).

PHB-PLA-Plasticizer	Hull	Stringing	Elephant Foot	Text Details	Cylindrical Shapes
REF (PHB/PLA)	3	2	1	1	3
C2-EH	4	3	3	2	4
A2-EH	4	2	3	3	3
PEG-2-EH	4	5	3	4	5

**Table 7:** Subjective visual evaluation of 3D Benchies: other chosen signs related to the material properties. Subjective marking (1–5, where 1 is the best).

PHB-PLA-Plasticizer	Square Shapes	Overhang Surfaces	Horizontal Holes	Layers Visibility	Warping
REF (PHB-PLA)	2	4	3	3	3
C2-EH	2	3	3	5	3
A2-EH	2	2	3	4	3
PEG-2-EH	2	5	4	4	4

**Table 8:** Average marking (1–5, where 1 is the best) of subjective evaluation of 3D Benchies.

PHB-PLA-Plasticizer	Average Mark
REF (PHB-PLA)	2.5
C2-EH	3.2
A2-EH	2.9
PEG-2-EH	4

**Table 9:** MDSC and DSC parameters of PHB-PLA-plasticizer samples and non-plasticized reference.

Sample	MDSC Scan	First Cooling DSC Scan		Second Heating DSC Scan		
	*T*_g, PLA_ (°C)	*T*_c_ (°C)	*H*_c_ (J/g)	*T*_m_ (°C)	*H*_m_ (J/g)	*X*_c_ (%)
REF	58.4	98	54.6	172	64.0	62.6
C2-EH	52.2	79	43.0	169	48.0	54.8
A2-EH	54.3	86	45.7	168	57.6	65.8
PEG-2-EH	21.7	81	32.0	169	47.9	54.7

**Table 10:** Young’s modulus (GPa) of PHB-PLA-plasticizer samples and of PHB-PLA reference.

Sample	Young’s Modulus (GPa)			
	Filaments—7 Days after Preparation	Filaments—40 Days after Preparation	Filaments—after 3 Days at 110 °C	Dogbones—7 Days after Printing
REF	0.57 ± 0.07	0.45 ± 0.12	0.59 ± 0.06	0.64 ± 0.21
C2-EH	0.34 ± 0.01	0.34 ± 0.03	Fragile-not measured	0.71 ± 0.11
A2-EH	0.33 ± 0.02	0.30 ± 0.01	0.31 ± 0.05	0.72 ± 0.08
PEG-2-EH	0.15 ± 0.01	0.18 ± 0.01	Fragile-not measured	0.56 ± 0.06

**Table 11:** Tensile strength (MPa) of PHB-PLA-plasticizer samples and of PHB-PLA reference.

Sample	Tensile Strength (MPa)			
	Filaments—7 Days after Preparation	Filaments—40 Days after Preparation	Filaments—after 3 Days at 110 °C	Dogbones—7 Days after Printing
REF	43.26 ± 1.00	42.86 ± 1.92	44.84 ± 0.49	40.90 ± 1.46
C2-EH	19.20 ± 0.76	18.92 ± 0.71	Fragile-not measured	26.11 ± 1.25
A2-EH	21.09 ± 0.70	18.72 ± 0.75	19.68 ± 1.38	26.74 ± 1.77
PEG-2-EH	15.11 ± 0.38	18.01 ± 0.58	Fragile-not measured	17.70 ± 0.73
